# 1-(Thio­phen-2-yl)-*N*-(4-{(*E*)-[(thio­phen-2-yl)meth­yl]imino­meth­yl}benzyl­idene)methanamine

**DOI:** 10.1107/S1600536812040809

**Published:** 2012-10-20

**Authors:** Haleden Chiririwa, John R. Moss, Denver Hendricks, Reinout Meijboom

**Affiliations:** a Department of Chemistry, University of Cape Town, Private Bag, Rondebosch, 7707, South Africa; b Division of Medical Biochemistry, Faculty of Health Sciences, Private Bag X3, Observatory 7935, South Africa; c Research Centre for Synthesis and Catalysis, Department of Chemistry, University of Johannesburg, PO Box 524 Auckland Park, Johannesburg, 2006, South Africa

## Abstract

The title compound C_18_H_16_N_2_S_2_, crystallizes with two independent half-mol­ecules in the asymmetric unit, in one of which the thio­phene rings are disordered in a 0.67:0.33 ratio. Each independent mol­ecule lies across a crystallographic centre of symmetry. The dihedral angle between central (half) benzene ring and the thiophene ring is 11.82°.

## Related literature

For similar thio­phenyl­dimine-based bridging ligands, see: Chakraborty *et al.* (1999 ▶); Haga & Koizumi (1985 ▶); Chiririwa *et al.* (2011*a* ▶,*b* ▶).
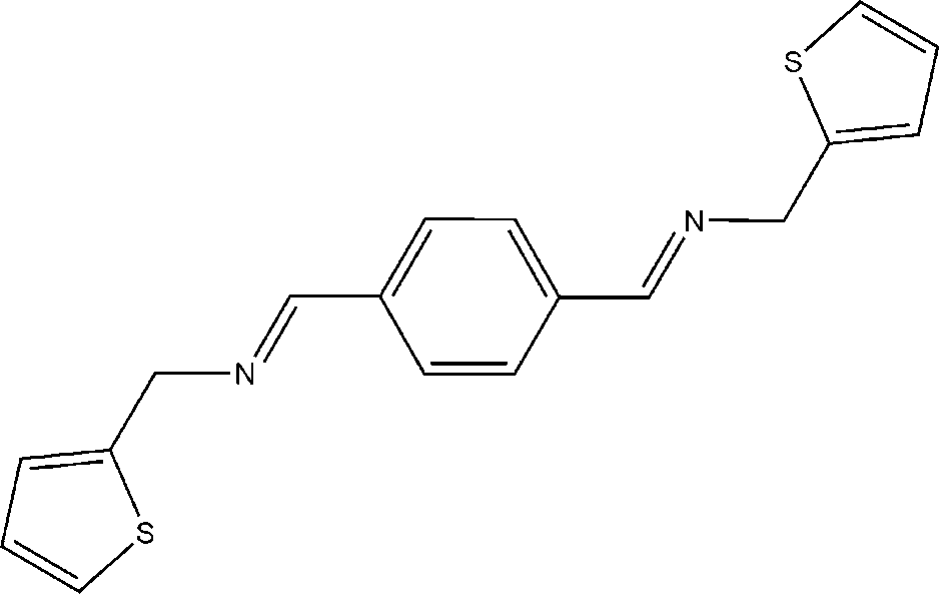


## Experimental

### 

#### Crystal data

C_18_H_16_N_2_S_2_
*M* *_r_* = 324.45
Triclinic, 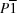

*a* = 8.8517 (3) Å
*b* = 10.3937 (5) Å
*c* = 10.5763 (4) Å
α = 63.836 (2)°
β = 69.023 (2)°
γ = 72.394 (2)°
*V* = 803.22 (6) Å^3^
*Z* = 2
Mo *K*α radiation
μ = 0.33 mm^−1^
*T* = 173 K
0.22 × 0.20 × 0.13 mm

#### Data collection

Nonius KappaCCD diffractometer
Absorption correction: multi-scan (*SADABS*; Bruker, 2007 ▶) *T*_min_ = 0.931, *T*_max_ = 0.959
34407 measured reflections
3282 independent reflections
2478 reflections with *I* > 2σ(*I*)
*R*_int_ = 0.052

#### Refinement

*R*[*F* ^2^ > 2σ(*F* ^2^)] = 0.045
*wR*(*F* ^2^) = 0.125
*S* = 1.06
3282 reflections
206 parameters
9 restraints
H-atom parameters constrained
Δρ_max_ = 0.34 e Å^−3^
Δρ_min_ = −0.48 e Å^−3^

### 

Data collection: *APEX2* (Bruker, 2007 ▶); cell refinement: *SAINT-Plus* (Bruker, 2007 ▶); data reduction: *SAINT-Plus* and *XPREP* (Bruker, 2007 ▶); program(s) used to solve structure: *SHELXS97* (Sheldrick, 2008 ▶); program(s) used to refine structure: *SHELXL97* (Sheldrick, 2008 ▶); molecular graphics: *DIAMOND* (Brandenburg & Putz, 2005 ▶), *ORTEP-3* (Farrugia, 1997 ▶); software used to prepare material for publication: *WinGX* (Farrugia, 1999 ▶).

## Supplementary Material

Crystal structure: contains datablock(s) I, global. DOI: 10.1107/S1600536812040809/go2071sup1.cif

Structure factors: contains datablock(s) I. DOI: 10.1107/S1600536812040809/go2071Isup2.hkl

Supplementary material file. DOI: 10.1107/S1600536812040809/go2071Isup3.cml

Additional supplementary materials:  crystallographic information; 3D view; checkCIF report

## Figures and Tables

**Fig. 1.**
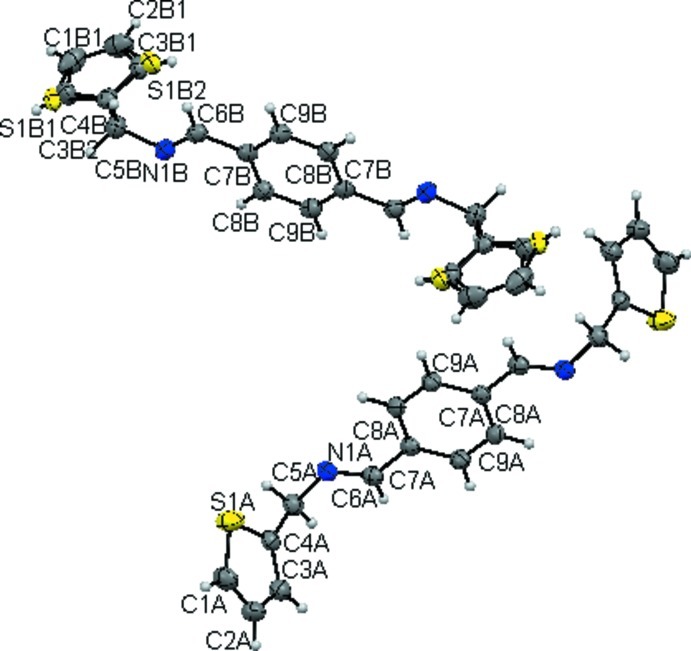
The structure of the (1,4-phenylenebis(methan-1-yl-1-ylidene)) bis(1-(thiophen-2-yl)methanamine) showing 40% probability displacement ellipsoids. Hydrogen atoms were omitted for clarity.

## supplementary crystallographic information

### Comment

The title compound belongs to a class of tetradentate ligands. To the best of our knowledge, this is the second example of a neutral thiophenyldimine-based bridging ligand, the first of which was reported earlier by our group (Chiririwa *et al.*, 2011*a*) and (Chiririwa *et al.*, 2011*b*). This compound is expected to chelate in a tetradentate manner with both nitrogen atoms coordinating along with the two thiophenyl sulphur atoms. Chakraborty *et al.* reported coordination of similar ligands to ruthenium (Chakraborty *et al.* 1999) whilst Haga and Kiozumi reported their coordination to molybednum, (Haga & Koizumi,1985). The bond lengths N1A—C6A = 1.265 (3)and N1B—C6B = 1.266 (3) Å are consistent with C=N double bonding.

### Experimental

A solution of benzene 1,4-dicarboxaldehyde (0.50 g, 3.73 mmol) in methanol (10 ml) was added dropwise to a stirred solution of 2-thiophenylmethylamine (1.20 g, 7.42 mmol) in methanol (10 ml). The mixture stirred at room temperature for *ca* 16 h. The precipitate was filtered off and washed with diethylether and dried under vacuum for 4 h affording a white powder in 85% yield. Crystals suitable for X-ray determination were obtained by recrystallization from CH_2_Cl_2_– hexane mixture at room temperature.: Calc. for C_18_H_16_N_2_S_2_: C, 66.63%; H, 4.97%; N, 8.63%; S, 19.77 Found: C, 66.59%; H, 4.62%; N, 8.72%; S, 19.65 1H NMR: (400 MHz) ?H 8.38 (t, 2H, J = 1.3 Hz) 7.82 (s, 2H) 7.25 (m, 4H) 7.00 (d, 4H, J = 3.4 Hz) 5.00 (d, 4H, J = 1.3 Hz. IR (KBr): 1612 cm-1 (C=N, imine)

### Refinement

The methine and aromatic H atoms were placed in geometrically idealized positions and constrained to ride on their parent atoms, with with C—H = 0.95 Å and *U*_iso_(H) = 1.2*U*_eq_(C) for aromatic, C—H = 0.99 Å and *U*_iso_(H) = 1.2*U*_eq_(C) for CH_2_ C—H = 0.95 Å and *U*_iso_(H) = 1.2*U*_eq_(C) for CH. Disorder refinement models were applied to the thiophenes of one independent molecule in the asymmetric unit. Geometrical (*FLAT*) restaraints were applied to keep the ring C1B1-C2B2-C3B1-C3B2-S1B1-S1B2 planar. Bond distance (*DFIX*) and 1,3 distance similarity restraints (*SADI*) were applied to obtain reasonable geometries. Ellipsoid displacement (*SIMU* and *DELU*) restraints were also applied to the disordered moieties. Free variables were connected to the disordered component to add to unity.

### Crystal data

**Table:**

C_18_H_16_N_2_S_2_	*Z* = 2
*M**_r_* = 324.45	*F*(000) = 340
Triclinic, *P*1	*D*_x_ = 1.341 Mg m^−^^3^
Hall symbol: -p 1	Mo *K*α radiation, λ = 0.71073 Å
*a* = 8.8517 (3) Å	Cell parameters from 34407 reflections
*b* = 10.3937 (5) Å	θ = 2.9–26.4°
*c* = 10.5763 (4) Å	µ = 0.33 mm^−^^1^
α = 63.836 (2)°	*T* = 173 K
β = 69.023 (2)°	Plate, colourless
γ = 72.394 (2)°	0.22 × 0.20 × 0.13 mm
*V* = 803.22 (6) Å^3^	

### Data collection

**Table:**

Nonius KappaCCD diffractometer	3282 independent reflections
Radiation source: fine-focus sealed tube	2478 reflections with *I* > 2σ(*I*)
Graphite monochromator	*R*_int_ = 0.052
1.2° φ scans and ω scans	θ_max_ = 26.4°, θ_min_ = 2.9°
Absorption correction: multi-scan (*SADABS*; Bruker, 2007)	*h* = 0→11
*T*_min_ = 0.931, *T*_max_ = 0.959	*k* = −11→13
34407 measured reflections	*l* = −11→13

### Refinement

**Table:**

Refinement on *F*^2^	Primary atom site location: structure-invariant direct methods
Least-squares matrix: full	Secondary atom site location: difference Fourier map
*R*[*F*^2^ > 2σ(*F*^2^)] = 0.045	Hydrogen site location: inferred from neighbouring sites
*wR*(*F*^2^) = 0.125	H-atom parameters constrained
*S* = 1.06	*w* = 1/[σ^2^(*F*_o_^2^) + (0.056*P*)^2^ + 0.4946*P*] where *P* = (*F*_o_^2^ + 2*F*_c_^2^)/3
3282 reflections	(Δ/σ)_max_ < 0.001
206 parameters	Δρ_max_ = 0.34 e Å^−^^3^
9 restraints	Δρ_min_ = −0.48 e Å^−^^3^

### Special details

**Table:**

Geometry. All e.s.d.'s (except the e.s.d. in the dihedral angle between two l.s. planes) are estimated using the full covariance matrix. The cell e.s.d.'s are taken into account individually in the estimation of e.s.d.'s in distances, angles and torsion angles; correlations between e.s.d.'s in cell parameters are only used when they are defined by crystal symmetry. An approximate (isotropic) treatment of cell e.s.d.'s is used for estimating e.s.d.'s involving l.s. planes.
Refinement. Refinement of *F*^2^ against ALL reflections. The weighted *R*-factor *wR* and goodness of fit *S* are based on *F*^2^, conventional *R*-factors *R* are based on *F*, with *F* set to zero for negative *F*^2^. The threshold expression of *F*^2^ > σ(*F*^2^) is used only for calculating *R*-factors(gt) *etc*. and is not relevant to the choice of reflections for refinement. *R*-factors based on *F*^2^ are statistically about twice as large as those based on *F*, and *R*- factors based on ALL data will be even larger.

### Fractional atomic coordinates and isotropic or equivalent isotropic displacement parameters (Å^2^)

**Table:**

	*x*	*y*	*z*	*U*_iso_*/*U*_eq_	Occ. (<1)
S1A	0.69140 (9)	−0.07229 (9)	0.71753 (8)	0.0551 (3)	
N1A	0.8877 (2)	0.1968 (2)	0.4771 (2)	0.0338 (5)	
C1A	0.4849 (3)	−0.0628 (3)	0.7957 (3)	0.0479 (7)	
H1A	0.4372	−0.1242	0.8918	0.058*	
C2A	0.3981 (3)	0.0419 (3)	0.7047 (3)	0.0431 (6)	
H2A	0.2815	0.0627	0.7295	0.052*	
C3A	0.4989 (3)	0.1187 (3)	0.5671 (3)	0.0383 (6)	
H3A	0.4571	0.1966	0.4905	0.046*	
C4A	0.6632 (3)	0.0676 (3)	0.5574 (3)	0.0322 (5)	
C5A	0.8063 (3)	0.1240 (3)	0.4339 (3)	0.0352 (6)	
H5A1	0.8850	0.0425	0.4092	0.042*	
H5A2	0.7678	0.1938	0.3465	0.042*	
C6A	0.8751 (3)	0.3337 (3)	0.4146 (3)	0.0312 (5)	
H6A	0.8204	0.3833	0.3389	0.037*	
C7A	0.9431 (3)	0.4184 (2)	0.4562 (2)	0.0284 (5)	
C8A	1.0455 (3)	0.3496 (3)	0.5499 (3)	0.0327 (5)	
H8A	1.0777	0.2468	0.5838	0.039*	
C9A	0.8997 (3)	0.5696 (3)	0.4064 (3)	0.0334 (5)	
H9A	0.8313	0.6177	0.3413	0.040*	
N1B	0.1122 (2)	0.6222 (2)	1.0089 (2)	0.0352 (5)	
C4B	0.3208 (3)	0.4419 (2)	0.9269 (3)	0.0335 (5)	
C5B	0.1887 (3)	0.4689 (3)	1.0535 (3)	0.0366 (6)	
H5B1	0.1048	0.4074	1.0881	0.044*	
H5B2	0.2367	0.4426	1.1349	0.044*	
C6B	0.1358 (3)	0.6967 (3)	1.0657 (3)	0.0324 (5)	
H6B	0.2009	0.6504	1.1335	0.039*	
C7B	0.0661 (3)	0.8528 (3)	1.0305 (3)	0.0316 (5)	
C8B	−0.0294 (3)	0.9267 (3)	0.9307 (3)	0.0327 (5)	
H8B	−0.0496	0.8767	0.8833	0.039*	
C9B	0.0946 (3)	0.9276 (3)	1.0991 (3)	0.0332 (5)	
H9B	0.1595	0.8779	1.1670	0.040*	
S1B1	0.34911 (15)	0.29538 (12)	0.88477 (13)	0.0351 (3)	0.67
C1B1	0.5072 (4)	0.3432 (3)	0.7441 (3)	0.0555 (8)	
H1B1	0.5647	0.2863	0.6858	0.067*	
C2B1	0.5492 (3)	0.4673 (3)	0.7172 (3)	0.0534 (8)	
H2B1	0.6371	0.5103	0.6402	0.064*	
C3B1	0.4387 (8)	0.5249 (7)	0.8248 (6)	0.0351 (3)	0.67
H3B1	0.4460	0.6137	0.8260	0.042*	0.67
S1B2	0.4503 (4)	0.5553 (3)	0.8214 (4)	0.0443 (7)	0.33
C3B2	0.3679 (13)	0.3308 (11)	0.8727 (12)	0.0443 (7)	0.33
H3B2	0.3115	0.2505	0.9179	0.053*	0.33

### Atomic displacement parameters (Å^2^)

**Table:**

	*U*^11^	*U*^22^	*U*^33^	*U*^12^	*U*^13^	*U*^23^
S1A	0.0367 (4)	0.0570 (5)	0.0504 (5)	−0.0094 (3)	−0.0160 (3)	0.0026 (4)
N1A	0.0311 (11)	0.0349 (11)	0.0371 (11)	−0.0090 (9)	−0.0081 (9)	−0.0134 (9)
C1A	0.0417 (15)	0.0519 (17)	0.0424 (16)	−0.0179 (13)	−0.0059 (12)	−0.0085 (13)
C2A	0.0320 (13)	0.0457 (15)	0.0534 (17)	−0.0068 (11)	−0.0105 (12)	−0.0204 (13)
C3A	0.0357 (13)	0.0390 (14)	0.0441 (15)	−0.0068 (11)	−0.0132 (11)	−0.0167 (12)
C4A	0.0354 (13)	0.0311 (12)	0.0367 (13)	−0.0079 (10)	−0.0115 (10)	−0.0155 (10)
C5A	0.0368 (13)	0.0357 (13)	0.0384 (14)	−0.0084 (11)	−0.0099 (11)	−0.0171 (11)
C6A	0.0295 (12)	0.0350 (13)	0.0275 (12)	−0.0078 (10)	−0.0066 (9)	−0.0097 (10)
C7A	0.0260 (11)	0.0302 (12)	0.0252 (11)	−0.0086 (9)	−0.0034 (9)	−0.0073 (9)
C8A	0.0319 (12)	0.0281 (12)	0.0344 (13)	−0.0068 (10)	−0.0098 (10)	−0.0064 (10)
C9A	0.0324 (12)	0.0332 (13)	0.0317 (13)	−0.0070 (10)	−0.0135 (10)	−0.0049 (10)
N1B	0.0316 (11)	0.0335 (11)	0.0402 (12)	−0.0020 (9)	−0.0094 (9)	−0.0160 (9)
C4B	0.0346 (13)	0.0326 (13)	0.0354 (13)	−0.0008 (10)	−0.0162 (10)	−0.0123 (10)
C5B	0.0391 (14)	0.0308 (12)	0.0384 (14)	−0.0037 (10)	−0.0112 (11)	−0.0125 (11)
C6B	0.0275 (12)	0.0340 (13)	0.0324 (13)	−0.0038 (10)	−0.0063 (10)	−0.0118 (10)
C7B	0.0252 (11)	0.0348 (13)	0.0322 (13)	−0.0048 (10)	−0.0033 (9)	−0.0137 (10)
C8B	0.0313 (12)	0.0356 (13)	0.0330 (13)	−0.0078 (10)	−0.0049 (10)	−0.0158 (10)
C9B	0.0287 (12)	0.0367 (13)	0.0328 (13)	−0.0047 (10)	−0.0079 (10)	−0.0126 (10)
S1B1	0.0441 (6)	0.0294 (6)	0.0375 (5)	−0.0095 (4)	−0.0131 (4)	−0.0136 (5)
C1B1	0.0624 (19)	0.0591 (19)	0.0555 (19)	0.0135 (15)	−0.0272 (16)	−0.0368 (16)
C2B1	0.0373 (15)	0.067 (2)	0.0419 (16)	−0.0066 (14)	−0.0023 (12)	−0.0158 (14)
C3B1	0.0441 (6)	0.0294 (6)	0.0375 (5)	−0.0095 (4)	−0.0131 (4)	−0.0136 (5)
S1B2	0.0408 (12)	0.0379 (16)	0.0556 (13)	−0.0146 (10)	−0.0074 (10)	−0.0177 (11)
C3B2	0.0408 (12)	0.0379 (16)	0.0556 (13)	−0.0146 (10)	−0.0074 (10)	−0.0177 (11)

### Geometric parameters (Å, º)

**Table:**

S1A—C1A	1.708 (3)	C4B—C3B2	1.399 (5)
S1A—C4A	1.719 (3)	C4B—C5B	1.500 (3)
N1A—C6A	1.265 (3)	C4B—S1B2	1.640 (3)
N1A—C5A	1.473 (3)	C4B—S1B1	1.694 (2)
C1A—C2A	1.342 (4)	C5B—H5B1	0.9900
C1A—H1A	0.9500	C5B—H5B2	0.9900
C2A—C3A	1.424 (4)	C6B—C7B	1.476 (3)
C2A—H2A	0.9500	C6B—H6B	0.9500
C3A—C4A	1.372 (3)	C7B—C9B	1.394 (3)
C3A—H3A	0.9500	C7B—C8B	1.396 (3)
C4A—C5A	1.498 (3)	C8B—C9B^ii^	1.381 (3)
C5A—H5A1	0.9900	C8B—H8B	0.9500
C5A—H5A2	0.9900	C9B—C8B^ii^	1.381 (3)
C6A—C7A	1.478 (3)	C9B—H9B	0.9500
C6A—H6A	0.9500	S1B1—C1B1	1.642 (3)
C7A—C8A	1.394 (3)	C1B1—C2B1	1.332 (3)
C7A—C9A	1.394 (3)	C1B1—C3B2	1.459 (5)
C8A—C9A^i^	1.380 (3)	C1B1—H1B1	0.9500
C8A—H8A	0.9500	C2B1—C3B1	1.435 (5)
C9A—C8A^i^	1.380 (3)	C2B1—S1B2	1.591 (3)
C9A—H9A	0.9500	C2B1—H2B1	0.9500
N1B—C6B	1.266 (3)	C3B1—H3B1	0.9500
N1B—C5B	1.461 (3)	C3B2—H3B2	0.9500
C4B—C3B1	1.384 (5)		
			
C1A—S1A—C4A	92.37 (13)	C5B—C4B—S1B1	123.56 (17)
C6A—N1A—C5A	117.3 (2)	S1B2—C4B—S1B1	115.94 (16)
C2A—C1A—S1A	111.7 (2)	N1B—C5B—C4B	109.8 (2)
C2A—C1A—H1A	124.1	N1B—C5B—H5B1	109.7
S1A—C1A—H1A	124.1	C4B—C5B—H5B1	109.7
C1A—C2A—C3A	113.0 (2)	N1B—C5B—H5B2	109.7
C1A—C2A—H2A	123.5	C4B—C5B—H5B2	109.7
C3A—C2A—H2A	123.5	H5B1—C5B—H5B2	108.2
C4A—C3A—C2A	112.4 (2)	N1B—C6B—C7B	122.5 (2)
C4A—C3A—H3A	123.8	N1B—C6B—H6B	118.8
C2A—C3A—H3A	123.8	C7B—C6B—H6B	118.8
C3A—C4A—C5A	128.2 (2)	C9B—C7B—C8B	119.3 (2)
C3A—C4A—S1A	110.46 (19)	C9B—C7B—C6B	119.5 (2)
C5A—C4A—S1A	121.24 (18)	C8B—C7B—C6B	121.2 (2)
N1A—C5A—C4A	109.30 (19)	C9B^ii^—C8B—C7B	120.0 (2)
N1A—C5A—H5A1	109.8	C9B^ii^—C8B—H8B	120.0
C4A—C5A—H5A1	109.8	C7B—C8B—H8B	120.0
N1A—C5A—H5A2	109.8	C8B^ii^—C9B—C7B	120.7 (2)
C4A—C5A—H5A2	109.8	C8B^ii^—C9B—H9B	119.7
H5A1—C5A—H5A2	108.3	C7B—C9B—H9B	119.7
N1A—C6A—C7A	121.8 (2)	C1B1—S1B1—C4B	94.00 (13)
N1A—C6A—H6A	119.1	C2B1—C1B1—C3B2	102.2 (3)
C7A—C6A—H6A	119.1	C2B1—C1B1—S1B1	115.4 (2)
C8A—C7A—C9A	118.8 (2)	C2B1—C1B1—H1B1	122.3
C8A—C7A—C6A	121.2 (2)	C3B2—C1B1—H1B1	135.5
C9A—C7A—C6A	120.0 (2)	S1B1—C1B1—H1B1	122.3
C9A^i^—C8A—C7A	120.2 (2)	C1B1—C2B1—C3B1	108.1 (3)
C9A^i^—C8A—H8A	119.9	C1B1—C2B1—S1B2	119.7 (2)
C7A—C8A—H8A	119.9	C1B1—C2B1—H2B1	126.0
C8A^i^—C9A—C7A	121.1 (2)	C3B1—C2B1—H2B1	126.0
C8A^i^—C9A—H9A	119.5	S1B2—C2B1—H2B1	114.3
C7A—C9A—H9A	119.5	C4B—C3B1—C2B1	115.3 (4)
C6B—N1B—C5B	116.7 (2)	C4B—C3B1—H3B1	122.3
C3B1—C4B—C3B2	97.2 (4)	C2B1—C3B1—H3B1	122.3
C3B1—C4B—C5B	129.2 (3)	C2B1—S1B2—C4B	94.97 (16)
C3B2—C4B—C5B	133.6 (3)	C4B—C3B2—C1B1	117.2 (5)
C3B2—C4B—S1B2	105.9 (3)	C4B—C3B2—H3B2	121.4
C5B—C4B—S1B2	120.50 (18)	C1B1—C3B2—H3B2	121.4
C3B1—C4B—S1B1	107.2 (3)		
			
C4A—S1A—C1A—C2A	−0.1 (2)	C3B1—C4B—S1B1—C1B1	0.7 (4)
S1A—C1A—C2A—C3A	−0.2 (3)	C3B2—C4B—S1B1—C1B1	−2 (4)
C1A—C2A—C3A—C4A	0.5 (3)	C5B—C4B—S1B1—C1B1	−179.4 (2)
C2A—C3A—C4A—C5A	−177.0 (2)	S1B2—C4B—S1B1—C1B1	−0.5 (2)
C2A—C3A—C4A—S1A	−0.6 (3)	C4B—S1B1—C1B1—C2B1	−0.5 (3)
C1A—S1A—C4A—C3A	0.4 (2)	C4B—S1B1—C1B1—C3B2	2 (3)
C1A—S1A—C4A—C5A	177.1 (2)	C3B2—C1B1—C2B1—C3B1	−0.3 (9)
C6A—N1A—C5A—C4A	−110.5 (2)	S1B1—C1B1—C2B1—C3B1	0.2 (5)
C3A—C4A—C5A—N1A	108.5 (3)	C3B2—C1B1—C2B1—S1B2	0.9 (7)
S1A—C4A—C5A—N1A	−67.7 (2)	S1B1—C1B1—C2B1—S1B2	1.5 (4)
C5A—N1A—C6A—C7A	176.1 (2)	C3B2—C4B—C3B1—C2B1	−0.2 (8)
N1A—C6A—C7A—C8A	10.0 (3)	C5B—C4B—C3B1—C2B1	179.4 (3)
N1A—C6A—C7A—C9A	−167.3 (2)	S1B2—C4B—C3B1—C2B1	172 (4)
C9A—C7A—C8A—C9A^i^	0.9 (4)	S1B1—C4B—C3B1—C2B1	−0.7 (7)
C6A—C7A—C8A—C9A^i^	−176.4 (2)	C1B1—C2B1—C3B1—C4B	0.4 (7)
C8A—C7A—C9A—C8A^i^	−0.9 (4)	S1B2—C2B1—C3B1—C4B	−174 (3)
C6A—C7A—C9A—C8A^i^	176.4 (2)	C1B1—C2B1—S1B2—C4B	−1.5 (4)
C6B—N1B—C5B—C4B	−112.6 (2)	C3B1—C2B1—S1B2—C4B	4 (2)
C3B1—C4B—C5B—N1B	42.8 (6)	C3B1—C4B—S1B2—C2B1	−6 (3)
C3B2—C4B—C5B—N1B	−137.8 (9)	C3B2—C4B—S1B2—C2B1	1.4 (8)
S1B2—C4B—C5B—N1B	44.0 (3)	C5B—C4B—S1B2—C2B1	−179.9 (2)
S1B1—C4B—C5B—N1B	−137.1 (2)	S1B1—C4B—S1B2—C2B1	1.1 (3)
C5B—N1B—C6B—C7B	179.9 (2)	C3B1—C4B—C3B2—C1B1	−0.1 (11)
N1B—C6B—C7B—C9B	178.8 (2)	C5B—C4B—C3B2—C1B1	−179.6 (5)
N1B—C6B—C7B—C8B	−0.5 (4)	S1B2—C4B—C3B2—C1B1	−1.2 (13)
C9B—C7B—C8B—C9B^ii^	0.0 (4)	S1B1—C4B—C3B2—C1B1	177 (5)
C6B—C7B—C8B—C9B^ii^	179.3 (2)	C2B1—C1B1—C3B2—C4B	0.3 (12)
C8B—C7B—C9B—C8B^ii^	0.0 (4)	S1B1—C1B1—C3B2—C4B	−178 (4)
C6B—C7B—C9B—C8B^ii^	−179.3 (2)		

Symmetry codes: (i) −*x*+2, −*y*+1, −*z*+1; (ii) −*x*, −*y*+2, −*z*+2.

## References

[bb1] Brandenburg, K. & Putz, H. (2005). *DIAMOND* Crystal Impact GbR, Bonn, Germany.

[bb2] Bruker (2007). *APEX2*, *SAINT-Plus*, *XPREP* and *SADABS* Bruker AXS Inc., Madison, Wisconsin, USA.

[bb3] Chakraborty, S., Munshi, P. & Lahiri, G. K. (1999). *Polyhedron*, **18**, 1437–1444.

[bb4] Chiririwa, H., Meijboom, R. & Omondi, B. (2011*b*). *Acta Cryst.* E**67**, o922. 10.1107/S1600536811009810 PMC3099871 21754193

[bb5] Chiririwa, H., Moss, J. R., Su, H., Hendricks, D. & Meijboom, R. (2011*a*). *Acta Cryst.* E**67**, o921. 10.1107/S1600536811009809 PMC3099859 21754192

[bb6] Farrugia, L. J. (1997). *J. Appl. Cryst.* **30**, 565.

[bb7] Farrugia, L. J. (1999). *J. Appl. Cryst.* **32**, 837–838.

[bb8] Haga, M. & Koizumi, K. (1985). *Inorg. Chim. Acta*, **104**, 47–50.

[bb9] Sheldrick, G. M. (2008). *Acta Cryst.* A**64**, 112–122. 10.1107/S0108767307043930 18156677

